# Abundance alteration of nondominant species in fecal-associated microbiome of patients with SAPHO syndrome

**DOI:** 10.1186/s12866-021-02221-2

**Published:** 2021-05-30

**Authors:** Jianhua Zhen, Yuxiu Sun, Pengfei Zhao, Chen Li, Hesong Wang, Yini Li, Lu Zhao, Li Wang, Guangrui Huang, Anlong Xu

**Affiliations:** 1 School of Life Sciences, Beijing University of Chinese Medicine, Beijing, 100029 China; 2 Oncology Department, Dongfang Hospital, Beijing University of Chinese Medicine, Beijing, 100078 China; 3 Department of Traditional Chinese Medicine, Peking Union Medical College Hospital, Beijing, 100730 China; 4 Institute of Biology, Henan Academy of Sciences, Zhengzhou, 450008 Henan China

**Keywords:** Fecal-associated microbiome, SAPHO syndrome, Biomarkers

## Abstract

**Background:**

SAPHO syndrome is a group of symptoms consisting of synovitis, acne, pustulosis, hyperostosis and osteosis. There is no specific laboratory index assist in the diagnosis of SAPHO because of its highly heterogeneous clinical manifestations. Pathogenic microorganisms had been identified in biopsies of some SAPHO cases and particular gene mutations were also linked to the occurrence of SAPHO. It is largely unknown whether intestinal microbiome plays a role in pathogenesis of SAPHO. To explore the intestinal microbiome structure of SAPHO syndrome, fecal samples from 17 SAPHO patients and 14 healthy controls (HC) were collected for 16S rDNA sequencing.

**Results:**

Our results showed that there was no significant difference in alpha indexes and beta diversity between SAPHO and HC samples, while there were 14 operational taxonomic units (OTUs) in the Wilcoxon rank-sum test and 42 OTUs in the MetagenomeSeq analysis showed significant difference in distribution between the SAPHO and HC groups, 3 of which in Firmicutes were also observed in the random forest analysis and used to construct a receiver operating characteristic curve to evaluate the diagnostic value, the area under the curve was 0.86.

**Conclusion:**

Fecal-associated microbiome in the SAPHO samples was characterized by the alteration in abundance of some nondominant species, and the 3 selected OTUs in Firmicutes could serve as candidate biomarkers for SAPHO syndrome diagnosis.

**Supplementary Information:**

The online version contains supplementary material available at 10.1186/s12866-021-02221-2.

## Background

The incidence of SAPHO (synovitis, acne, pustulosis, hyperostosis and osteosis) syndrome reported in Europe was 1/100,000 with the highest incidence in women aged 20 to 50 [1, 2]. SAPHO syndrome has a prolonged and recurrent course and is often misdiagnosed and/or underrecognized because of its peculiar and heterogeneous clinical presentation. The main features of SAPHO syndrome consist of cutaneous and osteoarticular manifestations, the latter more often affects the anterior chest wall and has a typical radiologic finding called the “bull’s head sign” [3]. Currently, there are no validated diagnostic criteria or any specific laboratory indexes for SAPHO syndrome, consequently causing a delay in the diagnosis and treatment, increasing the suffering and economic burden for patients. Therefore, effective and specific molecular biomarkers are of great significance for SAPHO syndrome to improve the progress of diagnosis and evaluation.

The etiology and pathogenesis of SAPHO syndrome are still unclear; however, it was believed that SAPHO was genetic predisposition disease and that the occurrence of SAPHO involved mutations in multiple genes, such as *P53 (G72C)*, *MDM2 (T309G)*, *LPIN2* and *NOD2* [4, 5]. At the same time, *Propionibacterium acnes* (*P. acnes*, the most important as the most identified), *Staphylococcus aureus*, *Haemophilus parainfluenzae*, and *Actinomyces* were isolated from biopsies from different bone lesions or pustules in patients with SAPHO syndrome [6–8], suggesting that this disease might be secondary to some low pathogenic microorganisms. The abnormal expressions of cytokines and over-activation of immune cells, such as significant changes in IL-8, TNF-α, IL-23, IL-17 and NK cells, were also reported to accompany SAPHO syndrome [9–11]. Previous studies have shown that *P. acnes*, as a commensal bacterium on the skin, is a powerful trigger of NLRP3-inflammasome activation and IL-1β and TNF-α processing and secretion in monocytes-macrophages, leading to excessive inflammatory response in SAPHO patients [6, 12]. Thus, the occurrence of SAPHO syndrome is resulted from multiple factors including genetic factors, infections and immune dysregulation [6, 8].

The intestinal microbiome plays an important role in the regulation of the immune system, especially in immunocyte differentiation and inflammatory response. For instance, the intestinal microbiome and its metabolites could activate dendritic cells (DCs) and macrophages through pattern recognition receptors, such as Toll-like receptors (TLRs) and Takeda G-protein coupled receptor 5 (TGR5) on the surface of the cell membrane [13, 14]. Dendritic cells could further promote T cell differentiation through cytokines, for example, DC-secreted IL-23 could promote Th17 differentiation [13]. Macrophages could synthesize and secrete a variety of pro-inflammatory cytokines (such as IL-1β and TNF-α), leading to severe inflammation [15]. In the present study, we investigated the intestinal microbiome structure in the SAPHO syndrome with the fecal-associated microbiome (FAM) as a representative intestinal microbiome. We also screened specific operational taxonomic units (OTUs) as candidate biomarkers and diagnostic indicators for SAPHO syndrome to facilitate clinical diagnosis.

## Results

### Clinic features of the subjects in the study

Seventeen patients with SAPHO syndrome (herein referred to as SAPHO) and 14 healthy controls (herein referred to as HC) were recruited in this study (Table S1). No significant difference between the SAPHO and HC groups were observed in gender, age, concentration of hemoglobin (HGB) and platelet (PLT) counts, except for the white blood cell (WBC) counts (*P* = 0.049, Table S1). These results showed that the WBC counts in the SAPHO samples were significantly higher than those in the HC samples.

### Overall structure of fecal-associated microbiome communities

**Fig. 1:**
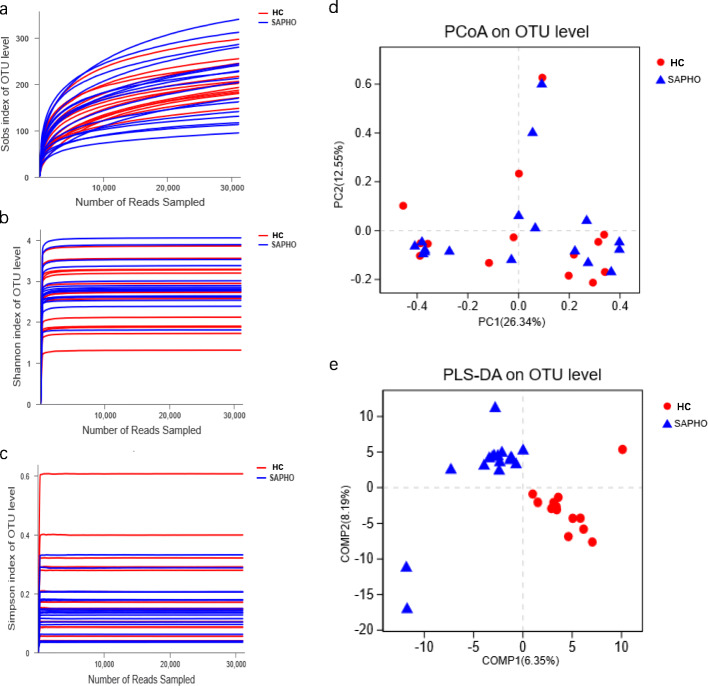
Comparison of alpha indexes and beta diversity in the HC and SAPHO samples. Rarefaction analysis of (**a**) Sobs index, (**b**) Shannon index and (**c**) Simpson index were performed to evaluate the bacterial diversity and richness, and plateaus of each curves indicated that the sequencing depth was enough. Different colors represent different groups. (**d**) PCoA based on Bray-Curtis distance metrics at the OTU level. (**e**) PLS-DA at the OTU level. HC, healthy control; SAPHO, SAPHO syndrome

In this study, 1,548,918 raw sequences from 31 fecal samples were generated, from which 1,253,798 high-quality sequences with an average length of 430 bp were obtained after quality control. Finally, an average of 40,445 reads per sample were recovered. After assigning to the SILVA 128/16S rDNA bacterial database, 994 OTUs that included 13 phyla, 26 classes, 65 orders, 118 families, 294 genera and 591 species, were detected. There was no significant difference between the SAPHO samples and the HC sampes in alpha indexes (such as the observed richness (Sobs), Simpson, Shannon, Chao 1 and Shannoneven indexes), which showed the bacterial diversity and richness (Table S2). However, the rarefaction curves of the Sobs, Shannon and Simpson indexes for all samples reached plateaus, indicating that no further sequencing were needed (Fig. 1a, b and c).

Principal coordinate analysis (PCoA) based on the Bray-Curtis distance metrics at the OTU level was employed to evaluate the bacterial communities, in which there was no obvious separation between the two groups (Fig. 1d). However, partial least squares discriminant analysis (PLS-DA) - a supervised analysis suitable for high-dimensional data - was performed, in which the SAPHO samples distributed separately from the HC samples (Fig. 1e).

### Common and distinct bacterial taxa in the analyzed groups

**Fig. 2:**
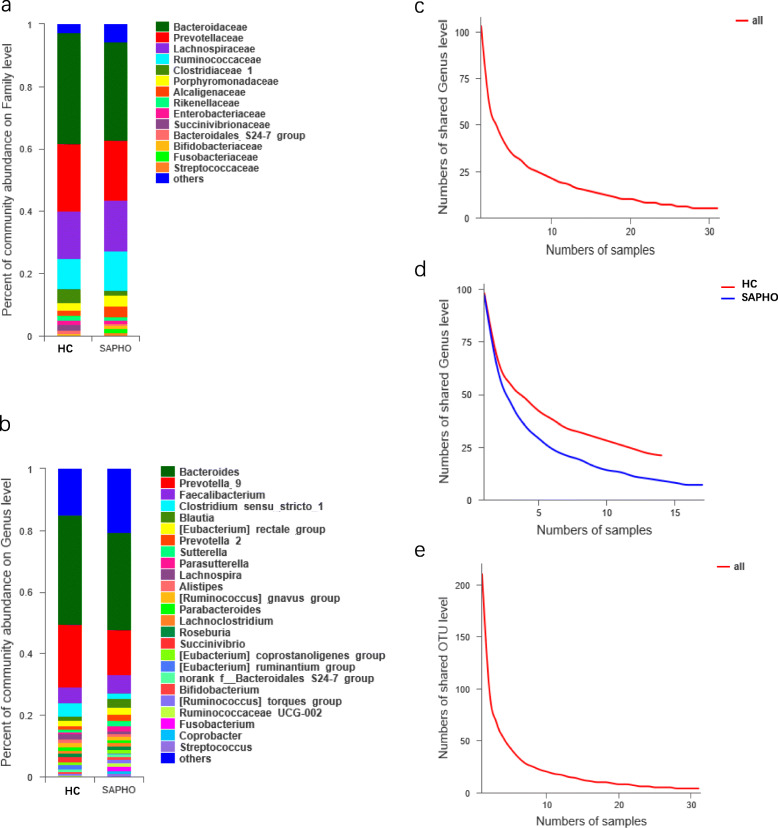
Relative abundances across samples at the family and genus levels, and core taxa analysis at the genus and OTU levels. Relative abundance of bacterial (**a**) families and (**b**) genera among the HC and SAPHO groups. Core genera analysis based on (**c**) all samples and (**d**) the HC/SAPHO samples respectively. (**e**) Core OTU analysis based on all samples. HC, healthy control; SAPHO, SAPHO syndrome

The relative abundances of predominant families/genera in SAPHO samples and HC samples were analyzed (Fig. 2). The top 5 most abundant families, including Bacteroidaceae, Prevotellaceae, Lachnospiraceae, Ruminococcaceae and Porphyromonadaceae, comprised 83.80% of all the taxa, in which the most abundant family was Bacteroidaceae with the relative abundance of 33.36% (Fig. 2a). Meanwhile, the top 5 most abundant genera included *Bacteroides, Prevotella, Faecalibacterium, Clostridium* *sensu stricto* *1* and *Blautia*, with the relative abundances of 33.36, 17.13, 5.64, 2.93 and 2.15%, respectively (Fig. 2b). Notably, only 5 genera were found in all samples, which represented more than 44.27% of all sequences, while 21 genera found in all HC samples and 7 found in all SAPHO samples (Fig. 2c and d). On the contrary of the average of approximate 208 OTUs per sample, only 4 OTUs were detected in all samples (Fig. 2e), which demonstrated the significant interindividual variation.

**Fig. 3:**
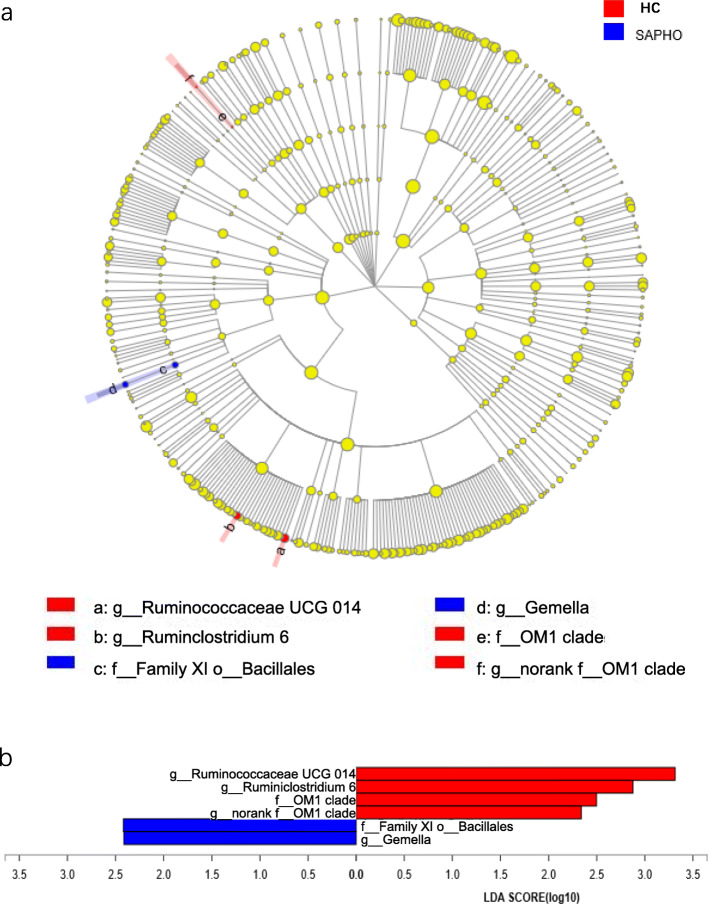
Distingushing taxa identified in the HC and SAPHO groups using LEfSe analysis. (**a**) Cladogram and (**b**) LDA score bar chart constructed using the LEfSe method. HC, healthy control; SAPHO, SAPHO syndrome

Consistent with the finding above, there were significant differences in bacterial composition between SAPHO samples and HC samples, which was supported by the results of MetagenomeSeq analysis, Wilcoxon rank-sum test and linear discriminant analysis (LDA) effect size (LEfSe) analysis (Tables S3 and S4; Fig. 3). In the MetagenomeSeq analysis, there were 42 OTUs to be found with different distribution between the SAPHO and HC groups, while there were 14 differently distributed OTUs in the Wilcoxon rank-sum test (Tables S3 and S4). In the LEfSe, distinguishing taxa between groups were identified, in which OM1 clade, *Ruminococcaceae UCG 014*, *Ruminiclostridium 6* and *norank f__OM1 clade* were significantly enriched in HC samples, while Family XI o__Bacillales and *Gemella* showed higher abundances in SAPHO samples (Fig. 3).

### Fecal-associated microbiome genera correlated with the clinical data in the SAPHO samples

**Fig. 4:**
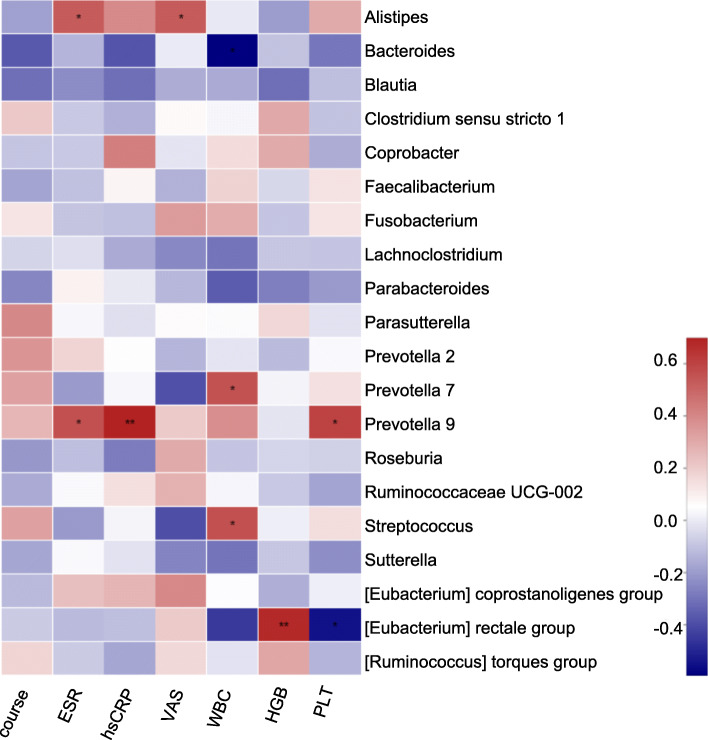
Associations of FAM genera with clinical data in 16 SAPHO samples. Pearson’s correlation values ranged from − 0.594 (blue) to 0.698 (red). ESR, erythrocyte sedimentation rate; hsCRP, high-sensitivity C-reactive protein; VAS, visual analogue score; WBC, white blood cell; HGB, hemoglobin; PLT, platelet

Additionally, the relationship between the top 20 genera and the clinical data in the 16 SAPHO samples (except SAPHO-11 due to the absence of visual analogue score (VAS)) was analyzed with Pearson’s correlations (Fig. 4). The strongest positive correlation was found between *Prevotella 9* and high-sensitivity C-reactive protein (hsCRP) (R = 0.698, *P* < 0.01), followed by *[Eubacterium] rectale group* and HGB (R = 0.681, *P* < 0.01), while the strongest negative correlation was found between *Bacteroides* and WBC counts (*R* = -0.594, *P* < 0.05), followed by *[Eubacterium] rectale group* and PLT (R = -0.551, *P* < 0.05). *Alistipes* and *Prevotella 9* were positively related to erythrocyte sedimentation rate (ESR) (*R* = 0.532, *P* < 0.05 and *R* = 0.564, *P* < 0.05), while *Alistipes* was the only positive correlation with VAS (*R* = 0.531, *P* < 0.05). In addition to *Bacteroides, Prevotella 7* and *Streptococcus* were also positively correlated with WBC counts (R = 0.559, *P* < 0.05 and *R* = 0.564, *P* < 0.05). Except for hsCRP, *Prevotella 9* was also positively correlated to ESR and PLT (*R* = 0.564, *P* < 0.05 and *R* = 0.603, *P* < 0.05).

### Random forest analysis and Wilcoxon rank-sum test

**Fig. 5:**
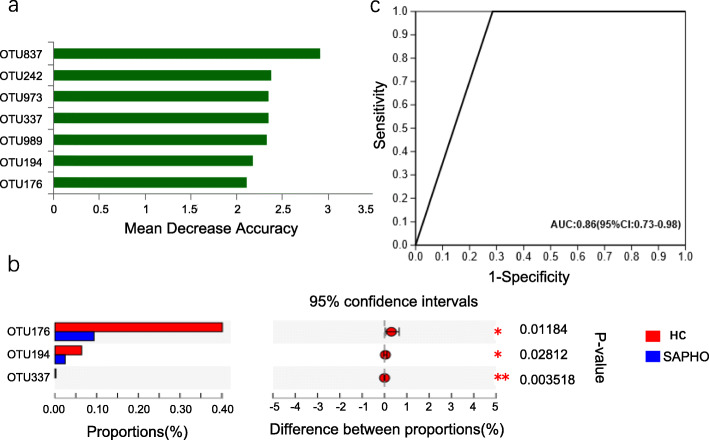
Random forest analysis and indicative OTUs as biomarkers of SAPHO in a ROC curve. (**a**) The top 7 OTUs in the random forest analysis and their mean decrease accuracy. (**b**) Indicative OTUs as biomarkers of SAPHO syndrome in Wilcoxon rank-sum test. (**c**) Three common indicative OTUs of random forest analysis and Wilcoxon rank-sum test were used to construct ROC curve to predict the diagnostic power. HC, healthy control; SAPHO, SAPHO syndrome

Random forest analysis provided further support for identifying the differential taxa between SAPHO and HC samples, which screened out an OTU-based signature that effectively distinguished the SAPHO patients from the healthy subjects. Using 10-fold-validation, the diagnostic model had 7 OTUs, 3 of which were also identified in Wilcoxon rank-sum test and significantly enriched in the HC samples (Fig. 5a, b and Table S5). Then, a receiver operating characteristic (ROC) curve was constructed based on these 3 OTUs, and the area under the curve (AUC) was 0.86 (Fig. 5c). Therefore, we speculated that these 3 OTUs could be used as FAM biomarkers of SAPHO syndrome. According to the random forest importance scores, these biomarkers included a member of the genus *Lachnospira*, a member of the genus *Faecalitalea* and a member of the species *[Eubacterium] siraeum DSM 15702* (Table S5), which were all in Firmicutes.

### Functional predictions

**Fig. 6:**
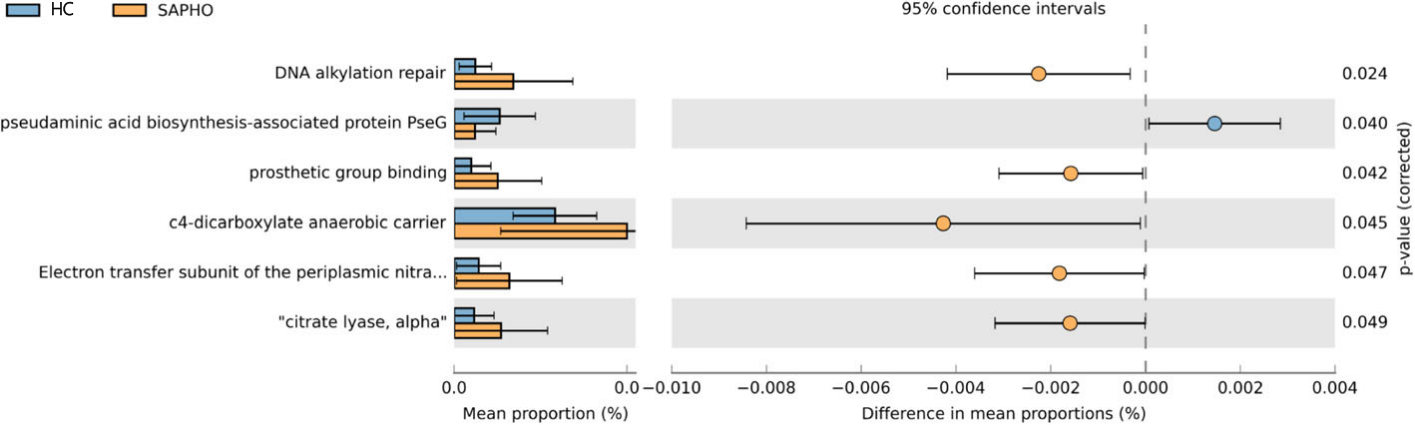
Wilcoxon rank-sum test outputs of predicted gene function enriched in different groups using PICRUSt. HC, healthy control; SAPHO, SAPHO syndrome

Finally, the bacterial function was predicted using the Phylogenetic Investigation of Communities by Reconstruction of Unobserved States (PICRUSt) algorithm, and the difference between the SAPHO and HC groups in Cluster of Orthologous Groups of proteins (COG) functions was exhibited in Wilcoxon rank-sum test (Fig. 6 and Table S6). Cluster of Orthologous Groups of proteins related to DNA alkylation repair (COG4335, *P* = 0.024), prosthetic group binding (COG3052, *P* = 0.042), C4-dicarboxylate anaerobic carrier (COG1288, *P* = 0.045), electron transfer subunit of the periplasmic nitrate reductase complex NapAB (By similarity) (COG3043, *P* = 0.047) and “citrate lyase, alpha” (COG3051, *P* = 0.049) were remarkably enriched in the SAPHO samples, while COG related to pseudaminic acid biosynthesis-associated protein PseG (COG3980, *P* = 0.040) was enriched in the HC samples (Fig. 6).

## Discussion

### Fecal-associated microbiome changed in the progress of SAPHO syndrome

Fecal-associated microbiome alteration is involved in the progresses of various diseases such as diabetes, metabolic syndrome, obesity, rheumatoid arthritis, and inflammatory bowel disease. However, whether FAM changed in SAPHO syndrome is still unclear, which is one of the purposes of this study.

In our study, the FAM structure in patients with SAPHO syndrome showed diversity and complexity as well as high variations among patients, which was also observed within the FAM structure of healthy control subjects. Notably, the distribution of dominant taxa at the family and genus levels was basically the same between the SAPHO and HC samples, which indicated that gut microbiota with high relative abundances might be indispensable for life-supporting functions (e.g., metabolism and immunity), and the specific species that could distinguish the two groups might be nondominant taxa with low abundances and might not have significant impacts on life. The speculation above was validated by the result of the LEfSe analysis, which showed 6 taxa with abundances less than 1% distributed differently between groups (Fig. 3), as well as that of Wilcoxon rank-sum test (Table S4). In addition, the abundances of OTUs based on the MetagenomeSeq results did not exceed 1% either, except OTU471 with an abundance of 1.420% in the SAPHO samples (Table S3). As these results shown, FAM changed in the progress of SAPHO syndrome, with an abundance alternation of some special nondominant species.

Considering the major roles that the dominant bacteria play in metabolism and immunity, we further explored the correlation between the top 20 genera and the clinical data of SAPHO samples. The main results showed that ESR was positively correlated with *Alistipes* and *Prevotella 9*, and hsCRP was positively correlated with *Prevotella 9* (Fig. 4). However, the abundances of these two genera were not significantly different between HC and SAPHO groups (Figure S1). The VAS, an important index for evaluating joint pain, was positively correlated with *Alistipes*; WBC counts, the representative of immunocytes in peripheral blood and systematic inflammation status, were positively correlated with *Prevotella 7* and *Streptococcus* while *Bacteroides* was negatively correlated. However, these correlations disappeared in all samples (Figure S2), although the variation trends of the abundances of the 3 genera between the SAPHO and HC samples were consistent with clinical expectation (Figure S1). In addition, the positive correlations between HGB and *[Eubacterium] rectale group* as well as PLT and *Prevotella 9* and the negative correlation between PLT and *[Eubacterium] rectale group* were also significant in all samples, and the variation trends of these 2 genera were opposite (Figure S1). These results suggest that the abundance changes of certain dominant bacteria may lead to changes in clinical indicators in patients with SAPHO syndrome, which can be used as biomarkers of clinical improvement; however, there are still some other dominant bacteria that do not show different distribution, for example, *Alistipes* and *Prevotella 9*, which are positively correlated with ESR, hsCRP and VAS, and enriched in the HC samples. This may be partly because of the small sample size included in this study, which cannot fully reflect the real clinical situation, and partly because of the heterogeneity of SAPHO syndrome. Further research is needed to collect more samples and clinical laboratory information from SAPHO patients for in-depth analysis.

### Specific OTUs as biomarkers of SAPHO syndrome to assist diagnosis

As FAM changed in SAPHO syndrome, it is possible that specific species that distributed differently between groups can be used as biomarkers of SAPHO syndrome to assist diagnosis. Three OTUs (OTU176, OTU194 and OTU337), selected by Wilcoxon rank-sum test and random forest analysis, were used to construct the ROC curve, and the AUC was 0.86, which showed excellent diagnostic significance and indicated that OTU176, OTU194 and OTU337 can function as clinical biomarkers. These 3 OTUs were all found in Firmicutes and decrease dramatically in the SAPHO samples; the taxa annotations were *g__Lachnospira*, *g__Faecalitalea* and *s__[Eubacterium] siraeum DSM 15702*.

*Lachnospira* can be detected in the microbiomes from the intestine, oral cavity and tongue fur [16, 17], which is a producer of short chain fatty acids (SCFAs) by catalyzing the fermentation of cellulose [18]. Short chain fatty acids could serve as energy source for intestinal mucosal epithelial cells and up-regulate tight junction proteins to maintain the integrity of mucosal mechanical barrier in intestine. *Lachnospira* can regulate intestinal local pH to maintain the stability of the chemical barrier. *Lachnospira* also triggered in the immune response of the intestinal mucosa via pattern recognition receptors (e.g., TLRs) and intervened in the expression of related cytokines. Thus, the decrease in *Lachnospira* may reduce the production of SCFAs, which can lead to immune dysfunction and then the development of SAPHO syndrome. Previous studies have reported that *Lachnospira* abundance was negatively correlated with fasting blood glucose and the homeostasis model assessment of insulin resistances [19], while a decrease in *Lachnospira* was presented in individuals with increasing weights during pregnancy and premature delivery caused by preterm premature rupture of membranes, phenylketonuria, hand-foot-mouth disease, asthma, children with type 1 diabetes, and elders with *Clostridioides difficile* infection [16, 20–24], but significantly increasing in longevities [25].

The functions of *Faecalitalea* and *[Eubacterium] siraeum DSM 15702* are not yet clear. However, studies had shown that *Faecalitalea* was significantly increased in DSS-induced colitis rat models [26], and Erysipelotrichaceae, the family including this genus, was believed to be a trigger of the NF-κB and STAT3 pathways in colitis [27]. Moreover, Erysipelotrichaceae was enriched in healthy controls when compared with type 1 diabetes patients, and its positive correlation with butyrate in fecal samples was strong in dogs [28, 29]. The whole genome sequence of *[Eubacterium] siraeum DSM 15702* is available online (https://bacteria.ensembl.org/_eubacterium_siraeum_dsm_15702_gca_000154325/Info/Annotation/#assembly), which includes 2703 coding genes, 66 noncoding genes (small noncoding genes) and 2769 gene transcripts. Notably, BspA-like proteins, which had been encoded in *[Eubacterium] siraeum DSM 15702*, have been shown to participate in innate immune regulation by interaction with TLRs [30]. Thus, a change in *[Eubacterium] siraeum DSM 15702* abundance may cause immune imbalance and induce SAPHO syndrome.

Functional prediction based on the 16S rDNA sequence showed that there were abnormalities in energy metabolism and DNA damage repair in patients with SAPHO syndrome, indicating that the FAM in SAPHO patients might undergo metabolism dysregulation, which might be reasons or results of enhanced immune response in SAPHO patients.

### Prospects

In this study, only 7 common genera appeared in all the SAPHO samples, accounting for less than 3% of the 275 genera, which indicated that the individual differences in FAM among SAPHO samples were great and might be related to the complex manifestations and classifications of the disease, as well as the small sample size. Further studies based on large sample size are needed to validate the efficiency of the 3 OTUs as biomarkers for SAPHO syndrome diagnosis.

## Conclusion

Fecal-associated microbiome structure in SAPHO syndrome was characterized by the alteration in abundance of some special nondominant species, and the 3 selected OTUs in Firmicutes (OTU176, OTU194 and OTU337) could serve as biomarkers of SAPHO syndrome to assist clinical diagnosis.

## Methods

### Subject recruitment and sample collection

All subjects were recruited from two hospitals (Peking Union Medical College Hospital and the Third Affiliated Hospital of Beijing University of Chinese Medicine) in Beijing, China. The subjects were between 15 and 65 years old and did not use any specific medicine (especially nonsteroidal anti-inflammatory drugs (NSAIDs), glucocorticoid, antirheumatic drugs, bisphosphonates or biological agents) within 3 months before enrollment. Additionally, the healthy controls should have medical reports without abnormal indexes or any chronic diseases (such as cancer or neurological disease) within 3 months, while patients with SAPHO syndrome should be diagnosed by diagnostic criteria updated at the 67th Annual Scientific Conference of the American Society of Rheumatology in 2012. Female subjects that were pregnant, breast-feeding or planning to become pregnant within 6 months should be excluded, as well as subjects with anemia, that participated in other clinical studies, that donated blood within 3 months, had a history of drug abuse, or any other situation that investigators considered inappropriate for inclusion. This study was approved by the Ethics Committee of the Peking Union Medical College Hospital and carried out in accordance with the Helsinki Declaration of 1975 as revised in 2000. Written informed consents were obtained from all subjects. All experiments were performed in accordance with the approved guidelines in previous study [31].

### 16S rDNA sequencing

The extraction of total genomic DNA and the PCR procedure of 16S rDNA v3-v4 variable regions were performed in accordance with the previous study [31] with primers 343F (5′-TACGGRAGGCAGCAG-3′) and 798R (5′-AGGGTATCTAATCCT-3′). Equal amounts of amplicons were used to build the sequencing library for Illumina MiSeq platform.

After merging in Fast Length Adjustment of Short Reads (FLASH) software (v1.2.11, https://ccb.jhu.edu/software/FLASH/index.shtml) [32], the sequences were clustered into OTUs at 97% similarity in Quantitative Insights Into Microbial Ecology (QIIME) software (v2.0, http://qiime.org/install/index.html) [33], and by assigning the representative sequence of each OTU to the SILVA 128/16S rDNA bacterial database with a 97% cutoff value in Bayesian approach, the taxonomic data (from phylum to species) were obtained from the RDP classifier [34].

Rarefaction curves from sampling-based OTU analysis were visualized to display the bacterial diversity and richness [35], including Sobs, Simpson, Shannon, Chao 1 and Shannoneven. These alpha indexes were further compared in Wilcoxon rank-sum test to exhibit the structural differences. PCoA based on Bray-Curtis distance metrics and PLS-DA were performed to visualize and compare the bacterial communities in different groups [36]. In addition, distinguishing taxa between groups were identified by MetagenomeSeq, Wilcoxon rank-sum test and the LEfSe analysis, and the LEfSe results were visualized with cladograms and taxonomic bar charts [36]. Combined with the random forest analysis, the specific OTUs were screened out, which were used to construct a ROC curve to determine the diagnostic values as biomarkers for SAPHO. Relationships between the top 20 genera and the clinical data were explored by calculating Pearson’s correlation coefficients and visualized as heatmap using the R package. Functional compositions of the bacterial communities were predicted using PICRUSt according to the COGs in NCBI (https://www.ncbi.nlm.nih.gov/genome) [37].

### Statistical analysis

The SPSS software package (v25.0, SPSS Inc., Chicago, USA) was used for statistical calculations. The independent samples T-test procedure was used to analyze the variables with a normal distribution in the Shapiro-Wilk test; while the Kruskal-Wallis test procedure was used to analyze the variables with a nonnormal distribution [38]. Additionally, the chi-square test was used to analyze the count data. *P* < 0.05 was considered as significance.

## Supplementary Information

**Additional file 1: Figure S1.** Wilcoxon rank-sum test outputs of 6 genera correlated to clinical data.

**Additional file 2: Figure S2.** Associations of FAM genera with clinical data in all samples. Pearson’s correlation values ranged from -0.506 (blue) to 0.624 (red). WBC, white blood cell; HGB, hemoglobin; PLT, platelet.

**Additional file 3: Table S1.** Characteristics of the subjects included in this study.

**Additional file 4: Table S2.** Alpha indexes between HC and SAPHO groups in Wilcoxon rank-sum test.

**Additional file 5: Table S3.** OTUs with significant difference between HC and SAPHO groups in MetagenomeSeq analysis.

**Additional file 6: Table S4.** OTUs with significant difference between HC and SAPHO groups in Wilcoxon rank-sum test.

**Additional file 7: Table S5.** OTUs as biomarkers of SAPHO syndrome and their taxonomy.

**Additional file 8: Table S6.** COG functions in SAPHO and HC samples by predicting the function of 16S rDNA gene.

## Data Availability

Data and materials are available from corresponding authors on reasonable request.

## Abbreviations

FAM: Fecal-associated microbiome
AUC: Area under the curve
COG: Cluster of Orthologous Groups of protein
DC: Dendritic cell
ESR: Erythrocyte sedimentation rate
FLASH: Fast Length Adjustment of Short Reads
HC: Healthy control
HGB: Hemoglobin
hsCRP: High-sensitivity C-reactive protein
IL-1: Interleukin 1
IL-8: Interleukin 8
IL-17: Interleukin 17
IL-23: Interleukin 23
LDA: Linear discriminant analysis
LEfSe: LDA effect size
NK: Natural Killer
NSAID: Nonsteroidal anti-inflammatory drug
OTU: Operational taxonomic unit
*P.acnes*: *Propionibacterium acnes*
PCoA: Principal coordinate analysis
PICRUSt: Phylogenetic Investigation of Communities by Reconstruction of Unobserved States
PLS-DA: Partial least squares discriminant analysis
PLT: Platelet
QIIME: Quantitative Insights Into Microbial Ecology
ROC: Receiver operating characteristic
Sob: Observed richness
TLR: Toll-like receptor
TNF-α: Tumor necrosis factor α
VAS: Visual analogue score
WBC: White blood cell
