# Supplementary figures and images for: Abundance alteration of nondominant species in fecal-associated microbiome of patients with SAPHO syndrome
Source: BMC Microbiol. 2021 May 30;21:161. doi: 10.1186/s12866-021-02221-2 (PMC8166064; doi:10.1186/s12866-021-02221-2)

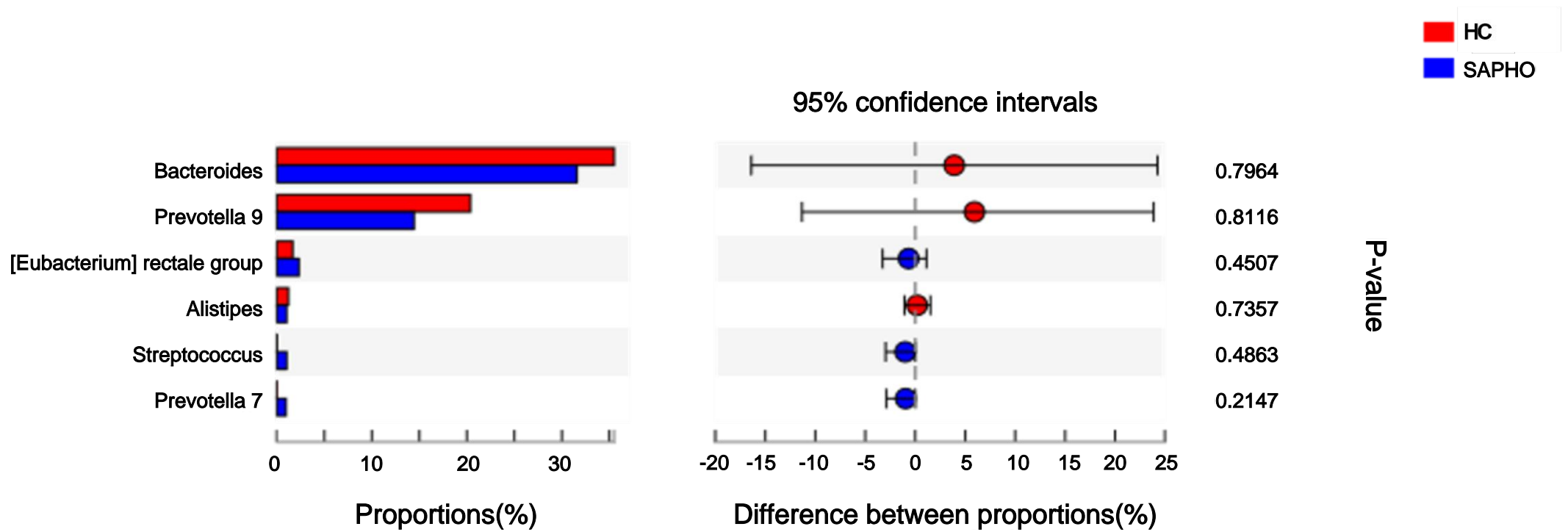

Supplement: Supplementary file 1 — Additional file 1: Figure S1. Wilcoxon rank-sum test outputs of 6 genera correlated to clinical data. [file 12866_2021_2221_MOESM1_ESM.pdf]

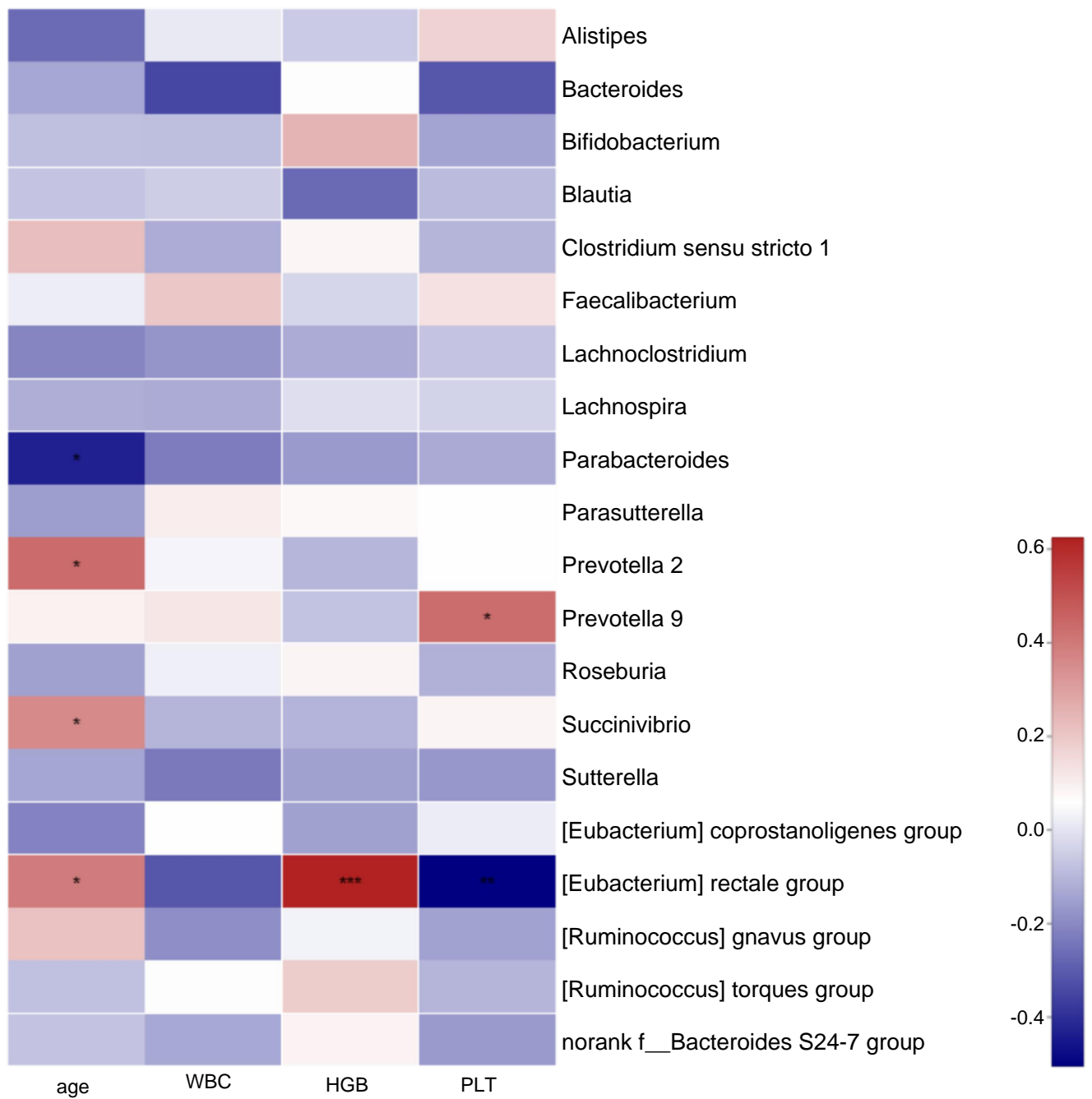

Supplement: Supplementary file 2 — Additional file 2: Figure S2. Associations of FAM genera with clinical data in all samples. Pearson’s correlation values ranged from -0.506 (blue) to 0.624 (red). WBC, white blood cell; HGB, hemoglobin; PLT, platelet. [file 12866_2021_2221_MOESM2_ESM.pdf]
